# Outcomes from the Northeast England cohort of autosomal dominant polycystic kidney disease (ADPKD) patients on tolvaptan

**DOI:** 10.3389/fneph.2022.984165

**Published:** 2022-09-23

**Authors:** Eleftherios Gkekas, Tsz Yau Tiffany Tang, Alan Green, Han Davidson, Rachel Fraser, John A. Sayer, Shalabh Srivastava

**Affiliations:** ^1^ Translational and Clinical Research Institute, Newcastle University, Newcastle upon Tyne, United Kingdom; ^2^ Renal Services, The Newcastle upon Tyne Hospitals National Health Service (NHS) Foundation Trust, Newcastle, United Kingdom; ^3^ National Institute for Health and Care Research (NIHR) Bioresource Centre, Newcastle upon Tyne, United Kingdom; ^4^ Nephrology Department, South Tyneside and Sunderland National Health Service (NHS) Foundation Trust, Sunderland, United Kingdom

**Keywords:** ADPKD (autosomal dominant polycystic kidney disease), tolvaptan treatment, end stage kidney disease (ESKD), disease progression, real world

## Abstract

Autosomal dominant polycystic kidney disease (ADPKD) is a cause of end-stage kidney disease (ESKD). The vasopressin V2-receptor antagonist tolvaptan has been shown within randomized clinical trials to slow down decline of kidney function in patients with ADPKD at risk of rapid progression. We performed a retrospective review of a Northeast England cohort of adult ADPKD patients who had been established on tolvaptan therapy to determine its efficacy in a real-world clinic setting. Other inclusion criteria involved a pre-treatment decline in greater than 2.5 ml/min/1.73m^2^/year based on readings for a 3 year period, and ability to tolerate and maintain tolvaptan treatment for at least 12 months. We calculated based on eGFR slopes, predicted time to reach ESKD with and without tolvaptan therapy. The cohort of patients included 21 from the Northeast of England. The mean rate of eGFR decline prior to treatment was -6.02 ml/min/1.73m^2^/year for the cohort. Following tolvaptan treatment, the average decline in eGFR was reduced to -2.47 ml/min/1.73m^2^/year, gaining a mean 8 years and 4 months delay to reach ESKD. The majority of patients (n=19) received and tolerated full dose tolvaptan (90 mg/30 mg). The real-life use of tolvaptan gave a dramatic improvement in eGFR slopes, much more than previously reported in clinical studies. These effects may be in part due to careful patient identification, selection and inclusion of patients who were able to tolerate tolvaptan therapy, excellent compliance with medication and a “tolvaptan clinic” effect where great personal care was given to these patients.

## Introduction

Autosomal dominant polycystic kidney disease (ADPKD) is the most common inherited kidney disorder leading to end stage kidney disease (ESKD) (1). The cystic change within the kidneys causes enlargement of the kidneys, leading to increases in total kidney volume (TKV), elevated blood pressure and a progressive decline in kidney function resulting in ESKD (2). Cysts may form in other organs including the liver, spleen and pancreas and there may be vascular abnormalities including cerebral aneurysms that may lead to significant morbidity and mortality (3).

ADPKD will most often present clinically in the 3^rd^ or 4^th^ decade of life with symptoms that include abdominal pain and fullness, infection of cysts, cyst hemorrhage with hematuria and hypertension (2).

ADPKD phenotypes are associated with a single heterozygous mutation in one of two main genes, *PKD1* and *PKD2* and a growing number of minor genes, including *IFT140* (4). Mutations in *PKD1* account for 85% of cases and are associated with more rapid disease progression (5).

The treatment of ADPKD patients traditionally relied upon supportive measures including blood pressure control, pain control, and prompt treatment of infected cysts, urinary tract infections and renal stones. Systemic blood pressure management was previously the only pharmacologically modifiable target based on evidence from clinical trials (6). In 2012, a treatment called tolvaptan, a vasopressin V2 receptor antagonist was shown to reduce both kidney growth and loss of estimated glomerular filtration rate (eGFR). This was based on the results of the Tolvaptan Efficacy and Safety in Management of Autosomal Dominant Polycystic Kidney Disease and Its Outcomes 3:4 Trial [TEMPO 3:4], a 3-year randomized double-blinded placebo controlled trial involving 1445 ADPKD patients (7). The effect of tolvaptan on eGFR was a slower decline in renal function as measured by reciprocal of serum creatinine level, compared with placebo. The decline in eGFR per year was -0.71 ml/min/1.73m^2^ per year in tolvaptan treated compared to -2.1 in historical controls (7).

In the United Kingdom, tolvaptan was approved by the National Institute of Clinical Excellence (NICE) in 2015 as the first drug to be used in the treatment ADPKD) (8). Since then, tolvaptan use has been advocated for ADPKD patients with evidence of rapid progression. Defining the patient cohort of rapid progression is subjective but the UK Renal Association provided some guidelines for its use in clinical practice (https://ukkidney.org/sites/renal.org/files/tolvaptan-in-adpkd-nice-commentary.pdf) as did the European Renal Association (9). A second study called REPRISE confirmed that tolvaptan was able to slow decline in eGFR on patients with later-stage ADPKD (10), with treatment effects being a gain of 2.36. ml/min/1.73m^2^ in patients with baseline eGFR of 45 to 59 following the 1 year study. Real world experience with the use of tolvaptan is now growing and the definitions of rapid progression have been clarified (8) allowing cohorts of patients separate from any clinical trials to be initiated and established on this therapy. Studies assessing long-term administration of tolvaptan for ADPKD convincingly show a sustained reduction of annual rate of eGFR decline (11).

Here, we present the outcomes from a Northeast England cohort of patients with ADPKD who were identified as being at risk of rapid progression and were commenced on tolvaptan within the renal outpatient department settings from two dedicated renal genetics clinics.

Within local patient databases, patients with ADPKD, who had features of rapid progression were identified and offered tolvaptan, as recommended by the UK Renal Association. This included patients over the age of 18 years with and established diagnosis of ADPKD, stage 2-3 CKD (30-89ml.min/1,73m^2^), a documented sustained decline in eGFR of ≥2.5 ml/min/1.73m^2^. Following these guidelines, we have used tolvaptan to treat ADPKD since its approval by NICE within dedicated renal genetics clinics serving the population of the Northeast England.

## Methods

This was a retrospective observational study to assess the impact of tolvaptan on the kidney function as measured by eGFR in patients with a clinical diagnosis of ADPKD. Patients underwent detailed phenotyping and long-term follow-up within the National Registry of Rare Kidney Diseases (RaDaR) (https://rarerenal.org/radar-registry/). We reviewed our cohort of patients on tolvaptan and included patients who met the following criteria:

1. Confirmed diagnosis of ADPKD (clinical/radiological findings consistent with the diagnosis and molecular genetic studies where available)
2. Age – 18-65 years
3. Estimated Glomerular filtration (eGFR) rate – 30-89 ml/min/1.73m^2^ as calculated by the CKD-EPI formula at the initiation of tolvaptan (12)
4. eGFR slope ≥-2.5 ml/min/1.73m^2^/year prior to initiation of tolvaptan using CKD-EPI formula and excluding values >90 ml/min/1.73m^2^. eGFR values at times of intercurrent illness and associated acute kidney injury were filtered out.
5. Tolvaptan therapy (at any dose) for greater than 18 months duration without treatment breaks/interruptions. The observation period for each patient was therefore at least 18 months. Throughout tolvaptan treatment patients would have an assessment of liver enzymes and eGFR on a 3 monthly basis.

We calculated the imaging classification of ADPKD patients using the Mayo Clinic algorithm (https://www.mayo.edu/research/documents/pkd-center-adpkd-classification/doc-20094754) by imputing total kidney volume (calculated by stereology measurements from kidney MRI or CT scan), patient height and patient age, resulting in Class 1A-1E. To plot eGFR slopes we retrospectively compared the slope of eGFR prior to tolvaptan initiation and following tolvaptan therapy. Specifically, prior to treatment, eGFR levels were collected from the first available levels up to three years before the date the patient was started on tolvaptan. The tolvaptan treatment eGFR levels were collected from the first available levels three months following the start date of tolvaptan therapy and up to four years after tolvaptan was started. eGFR levels taken within three months of starting tolvaptan were excluded to avoid the acute changes in eGFR upon drug commencement. The eGFR slopes were obtained using the function SLOPE() on Microsoft Excel on the raw data collected with the slope defined as the rate of change in eGFR over 1 year. We predicted time to ESKD (CKD stage 5, eGFR <15mls/min/1.73m^2^) for each patient based on the rate of eGFR decline. For graphical representation, we plotted the raw eGFR data in a scatter-plot graph for each patient, with the x-axis representing time (in years) and the y-axis representing eGFR level. A line of best fit was generated for pre-tolvaptan and tolvaptan eGFR values over time with confidence intervals of 95% added using a linear regression model (GraphPad Prism). Side effects including liver enzymes were assessed for each patient, overall bloods pressure control was reviewed and episodes of intercurrent illness noted.

## Results

**Figure 1:**
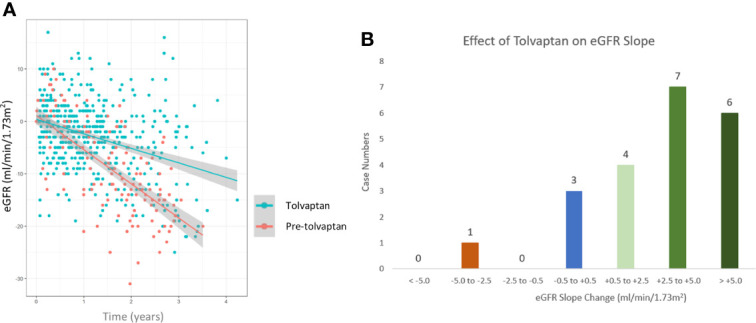
Comparison of estimated glomerular filtration (eGFR) slopes pre and post tolvaptan treatment in ADPKD patients **(A)** A scatter plot of eGFR (ml/min/1.73m^2^/year) over time in pre-tolvaptan and post-tolvaptan patients annotated with a line of best fit with 95% confidence intervals (CI) shown in grey shade. The rate of decline pre-tolvaptan was -6.51 ml/min/1.73m^2^ (slopes for 2.5% -7.53 & 97.5% -5.49) and the rate of decline on tolvaptan was -2.77 ml/min/1.73m^2^ (2.5% -3.39 & 97.5% -2.15) **(B)** The range of eGFR (ml/min/1.73m^2^/year) slope change and the number of patients in whom this was achieved in each range is shown.

**Figure 2:**
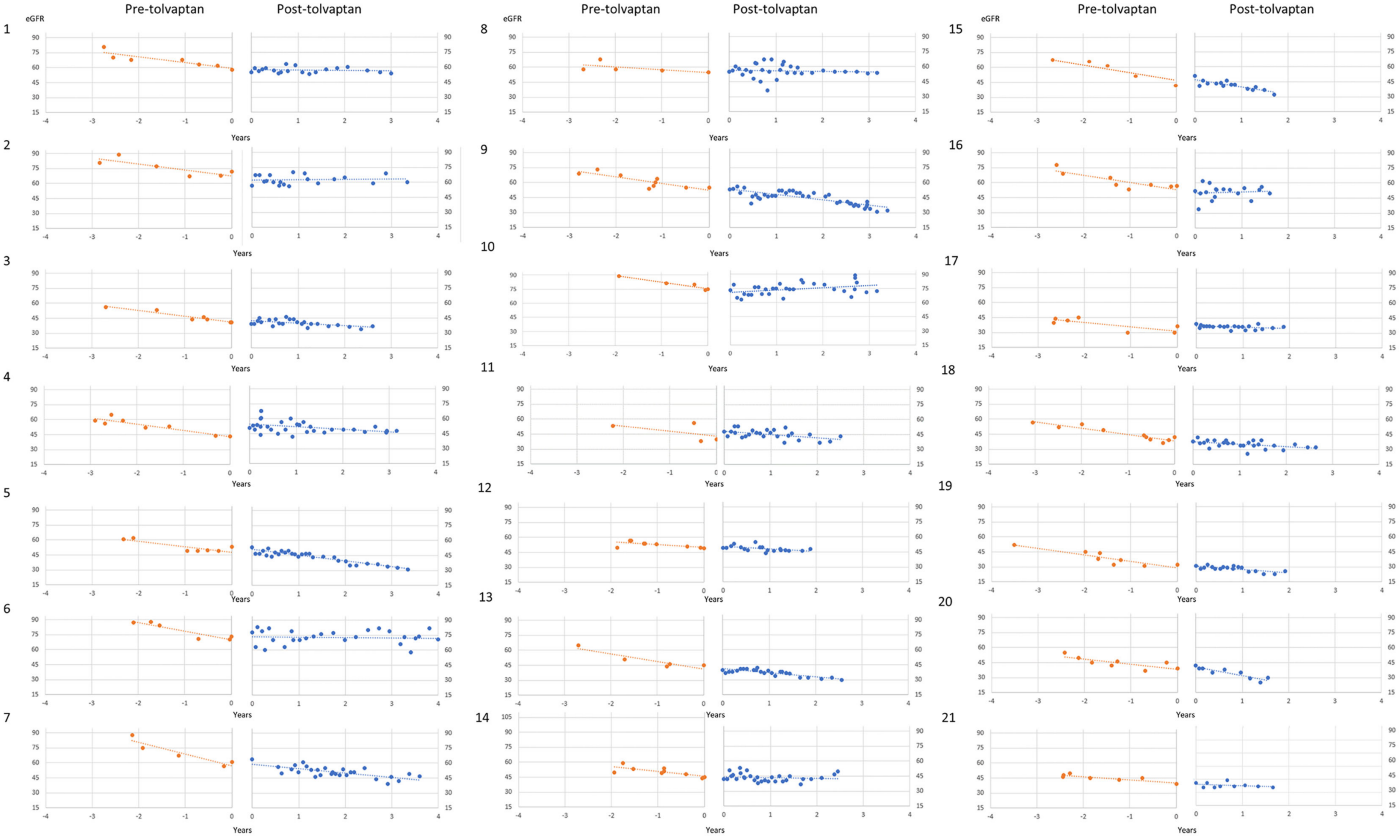
Individual patient eGFR plots pre- and post-tolvaptan eGFR (ml/min/1.73m^2^/year) is plotted against time in years for each of the patients.

A total of 21 patients with ADPKD who had been treated with tolvaptan met our inclusion criteria with a mean age of 48.0 years (range 30-62 years). There were 15 males and 6 females and all except 2 were on the maximal dose of tolvaptan (90 mg/30 mg) (**Table 1**). 14 patients had evidence of optimal blood pressure control below target of 130/80 and 7 had blood pressures above this target range. Two patients had intercurrent urinary tract infections (**Table 1**). The mean rate of eGFR decline prior to tolvaptan initiation was -6.02 ml/min/1.73m^2^ per year. This is consistent with this cohort having rapid progression of their disease (**Figure 1A**). Post tolvaptan initiation the eGFR decline was -2.47 ml/min/1.73m^2^ per year. Tolvaptan led to a mean change in the slope of decline by 3.56 ml/min/1.73m^2^ per year (**Figure 1A**). Individual patients and their eGFR plots demonstrate a wide range of responses (**Figure 2**). The majority of patients responded to treatment, with 1 who had a worsening of eGFR slope (ID 20) and 3 with an apparent non-response to the therapy with no change in eGFR slope (IDs 5, 12 and 15) (**Figure 1B**). The reasons for this lack of effect of response to tolvaptan are not fully apparent but it is noteworthy that 2 of these 4 cases had an ADPKD risk classification of 1E, the most severe category and that only 1 was less than 40 years of age.

**Table 1:** ADPKD patient baseline characteristics and tolvaptan dose.

Patient ID	Gender	Age (years)	eGFR (CKD-EPI) ml/min/1.73m^2^	ADPKD risk classification (MAYO score)	Molecular genetics	HTN <35 years of age	Urological Events <35 years	Tolvaptan dose (mg)	Blood Pressure	Acute events
1	M	58	58	1D	N/A	No	No	90/30	Raised	
2	M	39	72	1C	N/A	Yes	No	90/30	Controlled	
3	M	51	41	1C	*PKD1* frameshift	Yes	No	90/30	Raised	
4	M	48	43	1D	N/A	No	No	90/30	Raised	
5	M	51	53	1E	*PKD1* missense	Yes	No	90/30	Controlled	
6	F	54	73	1C	N/A	No	No	90/30	Controlled	
7	M	33	61	1E	*PKD1* splicing	Yes	No	90/30	Controlled	
8	M	52	55	1C	*PKD2* nonsense	No	No	90/30	Raised	
9	M	36	55	1E	N/A	Yes	No	90/30	Raised	
10	M	47	75	1B	N/A	Yes	No	90/30	Raised	
11	M	37	40	1D	N/A	Yes	Yes	90/30	Raised	
12	F	48	49	1C	*PKD1* nonsense	No	No	90/30	Controlled	UTI
13	M	45	45	1D	*PKD1* nonsense	No	No	90/30	Controlled	UTI
14	M	62	45	1D	*PKD2* missense	No	No	90/30	Controlled	
15	F	46	42	1C	*PKD1* nonsense	No	No	90/30	Controlled	
16	F	59	57	1D	N/A	No	No	90/30	Controlled	
17	M	54	36	1D	*PKD1* missense	No	No	90/30	Controlled	
18	F	47	42	1D	*PKD1* frameshift	Yes	No	90/30	Controlled	
19	F	52	32	1C	N/A	No	No	60/30	Controlled	
20	M	30	39	1E	N/A	Yes	Yes	90/30	Controlled	
21	M	58	76	1C	N/A	No	No	60/30	Controlled	

eGFR, estimated Glomerular Filtration Rate; F, Female; M, male; N/A, not available; UTI, urinary tract infection; Controlled BP control <130/80; Raised BP > 130/80

Prior to treatment, the mean predicted time to reaching ESKD in this cohort of ADPKD patients was 6.94 years. Following treatment with tolvaptan, the mean time to reach ESKD was 15.30 years. Tolvaptan therefore lead to a mean estimated delay of 8.36 (8 years and 4 months) years to reach ESKD in this cohort of rapidly progressing ADPKD patients.

## Discussion

ADPKD is the most common genetic condition leading to ESKD (13) and the most common life-threatening inherited disease worldwide. In recent years there have been significant gains in our understanding of the disease pathogenesis, its natural history and therapeutic interventions. The HALT-PKD trial (14, 15) demonstrated the benefits of strict blood pressure control and the effectiveness of blockade of the renin-angiotensin-aldosterone system in the reduction of progression of early ADPKD. The Consortium for Radiologic Imaging Studies of Polycystic Kidney Disease (CRISP) was a prospective, multicenter, observational study of ADPKD patients which determined the relationship between total kidney volume (TKV) and the decline of kidney function using cross sectional imaging. Now, baseline height-adjusted TKV can be used to predict decline of kidney function and progression to ESKD. Then randomized controlled studies TEMPO 3:4 (7) and REPRISE (10) introduced the use of a disease modifying drug, tolvaptan into clinical practice.

These data supporting the use of tolvaptan as a therapeutic intervention for ADPKD has been acquired from carefully designed multinational trials. The overall effect size on eGFR was relatively modest in both studies, suggesting that firstly patients were required to be on the drug for long periods of time to gain the most benefit and secondly that there may be significant variance in response rates to tolvaptan. The long-term gains of tolvaptan have been established in small cohorts of patients (11). A recent *post hoc* analysis of TEMPO 3:4 in Japanese ADPKD patients documented a change in eGFR of -8.5 ml/min/1.73m^2^ following tolvaptan treatment (compared to -12.6 in the placebo group) using a Japanese eGFR equation, suggesting that there may be population specific effects of this drug. A helpful update on the use of tolvaptan for ADPKD has recently been published which includes a treatment algorithm to guide patient selection (16).

We set out to assess its use in a real-world outpatient clinic setting by retrospectively reviewing our clinical service in the Northeast of England. Tolvaptan was found to be well tolerated in our patient cohort with the majority of patients receiving maximal dose of tolvaptan (120 mg per day). We excluded from this study patients who were unable to tolerate tolvaptan long term or had treatment breaks/holidays. We chose to monitor renal function using the CKD-EPI equation as this was the reported eGFR for our patients within our hospitals and represents real world data. We are aware of the inaccuracies in using eGFR equations in ADPKD patients. The CKD-EPI has an accuracy of 90%, and differences in accuracy were seen especially when the GFR was greater than 60 ml/min/1.73m^2^ (17).

We also chose to consider the slope of eGFR as a linear progression, however deviations from linearity (unrelated to acute events) can occur in a proportion of patients (18, 19). In an 10 year retrospective analysis of individual eGFR slopes, the majority of patients had a linear decline in eGFR, but some had more complex patterns including prolonged stable phases and negative exponential declines were seen, which are informative for clinical management, mechanistic studies and future clinical trial designs (19).

An alternative specific measure for ADPKD progression is serial renal volume measurement using MRI scanning (20, 21) but this was not routinely performed in our clinical practice and serial TKV measurement has not been recommended for the monitoring of response to tolvaptan (22). There is some evidence that greatest renal benefit is linked to the greatest suppression of urine osmolality (23), however we did not assess this in our cohort. We had no reports of deranged liver enzymes or other severe reactions. In our analysis, we demonstrate good overall efficacy of tolvaptan in our patient cohort whereby it led to a mean gain of > 10 ml/min/1.73m^2^ over a 3 year treatment period. The main strengths of our study are its “in practice”, clinic-based data which lends itself to real world application. In this regard we were able to define individual response rates to tolvaptan on eGFR slopes. There were 4 patients who failed to respond to tolvaptan or in whom it had no discernible effect on the decline in eGFR. Two of these patients had massively enlarged kidneys leading to an ADPKD prediction score of 1E. Of note, the measurement of eGFR whilst taking tolvaptan is reduced, due to the reversible inhibition if the tubulo-glomerular feedback mechanisms (24) and may have masked a small response in some of these cases. As this was a retrospective study, we did not include measurement of eGFR after a washout period. It may be important for future use of tolvaptan and other pharmacological agents to determine biomarkers of responsiveness. Measurement of urine osmolality can be used as a tool to assess compliance but may also provide a window into the effect of tolvaptan on the kidney. In the TEMPO 3:4 study baseline urinary osmolarity in ADPKD reflected age, kidney function and TKV and the greatest kidney benefit occurred in patients achieving greater suppression of urine osmolarity (23). Routine measurement of urinary osmolality before and after treatment might be a useful tool to guide the efficacy of tolvaptan. The main weaknesses of this study are the small sample size and retrospective data collection with inclusion of only patients who were able to tolerate the drug.

In summary, we report significant improvement in eGFR slope and time to CKD 5 in patients on Tolvaptan in our cohort but highlight that there are sub-groups of tolvaptan non-responders who may benefit from alternative approaches. The maximum dose of tolvaptan was well tolerated. The precise reasons for both non-response and exaggerated response need to be evaluated carefully to determine how individualization of tolvaptan therapy can be utilized for patient benefit.

## Data availability statement

The original contributions presented in the study are included in the article. Further inquiries can be directed to the corresponding author.

## Ethics statement

Ethical review and approval was not required for the study on human participants in accordance with the local legislation and institutional requirements. The patients/participants provided their written informed consent to participate in this study.

## Author contributions

JS and SS contributed to conception and design of the study. EG and TYTT organized the database and performed the analysis. SS wrote the first draft of the manuscript. EG, TYTT, AG, HD and RF wrote sections of the manuscript. All authors contributed to manuscript revision, read, and approved the submitted version.

## Funding

SS is funded by PKD Charity, ref. ADPKD-19-01. JAS is funded by PKD Charity, Kidney Research UK, ref Paed_RP_001_20180925 and the Northern Counties Kidney Research Fund, ref. 2020-01. We thank Dr Ian Wilson, Newcastle University for statistical support.

## Conflict of interest

The authors declare that the research was conducted in the absence of any commercial or financial relationships that could be construed as a potential conflict of interest.

## Publisher’s note

All claims expressed in this article are solely those of the authors and do not necessarily represent those of their affiliated organizations, or those of the publisher, the editors and the reviewers. Any product that may be evaluated in this article, or claim that may be made by its manufacturer, is not guaranteed or endorsed by the publisher.
